# Une étude rétrospective sur le cancer de l'ovaire avec un recul médian de 42 mois

**DOI:** 10.11604/pamj.2015.20.211.6113

**Published:** 2015-03-09

**Authors:** Fanomezantsoa Raherinantenaina, Solonirina Davidà Rakotomena, Nomeharisoa Rodrigue Emile Hasiniatsy, Felantsoa Auberlin Rakototiana, Florine Rafaramino, Hery Nirina Rakoto Ratsimba

**Affiliations:** 1 Service de Chirurgie Générale et Vasculaire, CHUJRA, Antananarivo Madagascar; 2 Service d'Oncologie-Hématologie-Radiothérapie, CHUJRA, Antananarivo Madagascar; 3 Service d'Urologie, CHUJRA, Antananarivo Madagascar

**Keywords:** Cancer, Chirurgie, Chimiothérapie, Ovaire, Cancer, surgery, Chimiotherapy, Ovary

## Abstract

Le cancer de l'ovaire est relativement fréquent mais grave et de mauvais pronostic. Le but de cette étude était de mettre en évidence les aspects épidémiologique, diagnostique, thérapeutique et évolutif de cette pathologie maligne prise en charge dans un pays en développement. Il s'agit d'une étude rétrospective et descriptive de 10 ans (2000- 2009) effectuée dans un CHU de chirurgie générale et d'Oncologie sur 62 patientes ayant développé un cancer de l'ovaire et opérées à visée curative. L’âge moyen des patientes était de 43 ans dont 53,23% avaient plus de 45 ans. Le dosage sanguin du CA-125 était positif chez 10 patientes sur 12. Les tumeurs étaient découvertes à l’échographie dans 87,10% des cas et à la laparotomie dans 12,90%. L'hystérectomie totale avec annexectomie bilatérale était l'intervention la plus pratiquée (64,52%). Les suites opératoires précoces étaient simples. Dix patientes étaient opérées de second regard (16,13%) pour des récidives locorégionales. Les tumeurs épithéliales étaient le type histologique le plus fréquent (93,55%) dont 79% au stade avancé (Ic-IV) et 21% au stade précoce (Ia-Ib). La chimiothérapie adjuvante était administrée chez 22,60% des patientes. Avec un recul médian de 42 mois, 29 patientes étaient perdues de vue. L’évolution était favorable dans 27,42% et dans 25,81% les décès se sont survenus en postopératoire tardif. Le cancer de l'ovaire n’était pas fréquent mais grave compte tenu des stades avancés et du taux élevé des décès postopératoires tardifs qui étaient largement observés chez les patientes privées d'une chimiothérapie adéquate.

## Introduction

Le cancer de l'ovaire est une pathologie peu fréquente mais grave et de mauvais pronostic. Il représente la quatrième cause de décès féminins par cancer. Sa particularité repose sur son caractère silencieux responsable d'un retard diagnostique et de difficulté thérapeutique surtout dans ses formes étendues [1]. Afin d'améliorer le pronostic des patientes par une prise en charge précoce, des méthodes de dépistage systématique incluant un dosage du CA 125 et une échographie endovaginale ont été proposées mais les résultats étaient décevants [2]. La prise en charge reste difficile dans les centres moyens équipés comme le notre. L'amélioration du pronostic parait compliquée puisque la majorité des cas diagnostiqués sont au stade localement avancé. A la lumière de notre étude et à travers une revue de la littérature, nous discutons les particularités épidémiologique, diagnostique, thérapeutique et évolutive de cette tumeur maligne prise en charge dans un pays en développement.

## Méthodes

Il s'agit d'une étude rétrospective de 10 ans menée dans un centre hospitalier universitaire de chirurgie générale et d'oncologie (CHU-HUJRA Antananarivo Madagascar). Une série consécutive de 82 patientes opérées pour tumeurs malignes de l'ovaire entre Janvier 2000 et 31 Décembre 2009 a été étudiée. La collecte des données était faite par un observateur indépendant. Parmi ces 82 patientes, 20 cas opérés dans d'autres centres ont été exclus par manque de données thérapeutiques chirurgicales. Soixante deux dossiers complets ont été retenus pour la réalisation de ce travail. La date de dernier recul était Janvier 2013. A cette date, 29 patientes ont été perdues de vue. Les données ont été recueillies à partir des cahiers d'observation, de l'hospitalisation, et des comptes rendus opératoires. Les informations obtenues ont été saisies dans une base de données sous forme de tableau Excel^®^, permettant la réalisation de l'analyse statistique au moyen du logiciel Statview^®^. Du fait des faibles effectifs des sous-groupes, les statistiques réalisées sur cette série ont été uniquement descriptives.

## Résultats

La série analysée incluait 62 patientes ayant un cancer de l'ovaire soit 0,90% des tumeurs malignes observées chez la femme durant la période d’étude. L’âge moyen des patientes était de 43 ans avec des extrêmes 13 et 76 ans. Dans 53,23% des cas, les patientes étaient âgées de 45 à 76 ans. Vingt deux patientes (35,48%) étaient multipares. Les autres (64,52%) étaient nullipares (n = 18) ou avaient eu moins de deux enfants (n = 22). La douleur pelvienne était présente chez 38 patientes (61,29%) et représentait le symptôme le plus fréquent devant l'augmentation de volume de l'abdomen par l'existence d'une ascite (32,26%) (Tableau 1). Les tumeurs étaient découvertes à l’échographie dans 54 cas (87,10%) et à la laparotomie dans 8 cas (12,90%). Les antécédents familiaux de cancer ont été mentionnés chez 12 patientes (19,35%) et dans 50 cas aucun antécédent pathologique n'a été trouvé (80,65%) (Tableau 2). Le bilan d'extension comportait essentiellement des examens échographiques de l'abdomen (40 cas: 64,51%) et radiographiques du thorax (32 cas: 51,61%). Une seule patiente pouvait bénéficier un scanner thoraco- abdomino-pelvien (TAP) (1,61%) et deux autres une urographie intraveineuse (3,23%). Quatre patientes n'avaient aucun examen d'imagerie. En préopératoire, une biopsie d'adénopathies inguinales avec analyse histologique était faite chez trois patientes. Les marqueurs tumoraux dosés étaient: CA-125 positif (>35UI) chez 10 patientes sur 12, Alpha Fœto-Protéine (AFP) négatif chez une patiente sur deux, Antigène Carcino-Embryonnaire (ACE) négatif chez une patiente et CA 19-9 négatif chez deux autres. La cytologie du liquide d'ascite était réalisée chez 5 patientes dont les résultats mettaient en évidence 3 cas d'adénocarcinome et 2 cas de carcinome épidermoïde. Au moment du diagnostic, la taille moyenne des tumeurs était de 8 cm (extrêmes, 4 et 25cm). La localisation ovarienne des lésions était unilatérale dans 44 cas (71%) et bilatérale dans 18 cas (29%). Dans 41,94% des cas (n = 26), il existait des lésions extensives macroscopiquement visibles dans le pelvis. Une patiente avait une métastase hépatique (1,61%) et deux autres ont développé une carcinose péritonéale sans atteinte hépatique (3,26%). Dans tous les cas (100%), la chirurgie était effectuée de façon conventionnelle à travers une laparotomie médiane xyphopubienne. L'hystérectomie totale avec annexectomie bilatérale était l'intervention la plus pratiquée (40 cas: 64,52%) suivie de l'annexectomie unilatérale (10 cas: 16,13%). La tumorectomie était conservatrice dans 6 cas (9,7%) et six autres ont subi une ovariectomie (Tableau 3).

**Tableau 1:** Fréquence des symptômes et signes cliniques.

Signes d'appel observes	Fréquence	Pourcentage (%)
Douleur pelvienne	38	61,29
Augmentation du volume abdominal (Ascite)	20	32,26
Masse pelvienne palpable	13	20,97
Métrorragie, ménorragie	11	17,74
Œdème des membres inférieurs	4	6,45
Fièvre	3	4,84
Asthénie, anorexie, amaigrissement, pâleur	6	9,68
Aménorrhée	2	3,24
Trouble de transit, vomissement	3	4,84

**Tableau 2:** Antécédents familiaux de cancer.

Antécédents de cancer dans la famille	Effectif	Pourcentage (%)
Cancer du tube digestif	4	6,45
Cancer gynécologique (Ovaire, utérus, sein)	6	9,68
Cancer de la prostate	1	1,61
Cancer du poumon	1	1,61
Aucun	50	80,65
**Total**	62	100

**Tableau 3:** Principaux types d'intervention chirurgicale effectués.

Types d'intervention	Effectif	Pourcentage (%)
Hystérectomie totale avec annexectomie bilatérale	40	64,52
Annexectomie unilatérale	10	16,13
Tumorectomie conservatrice	6	9,68
Ovariectomie bilatérale (+ Tumorectomie)	4	6,45
Ovariectomie unilatérale (+ Tumorectomie)	2	3,22
**Total**	62	100

Les gestes chirurgicaux complémentaires associant omentectomie, lymphadénectomie pelvienne et lomboaortique étaient réalisés dans 6 cas (9,68%). Les autres interventions comportaient des résections digestives (n = 9: 14,52%), splénectomie (n = 1: 1,61%) et cystectomie partielle (n = 3: 4,84%). Parmi les résections digestives, nous avons noté 2 cas de gastrectomie partielle, 3 cas de résection iléale et 4 cas de résection colorectale. Aucune péritonectomie n'a été réalisée. La chirurgie sans résidu était observée chez 17 patientes (27,42%). Dans tous les cas, les suites opératoires précoces étaient simples et aucun décès n’était survenu en per ou postopératoire immédiat. Dix patientes ont été opérées de second regard (16,13%) pour des récidives locorégionales. Après analyse anatomopathologique des pièces opératoires, les tumeurs épithéliales de l'ovaire étaient le type histologique le plus fréquemment observé (58 cas: 93,55%) devant les tumeurs conjonctivale (1,61%), mésenchymateuse (1,61%) et germinale (3,23%) (Tableau 4). Selon la Classification de FIGO, il existait 79.03% (n = 49) de cancers au stade avancé (Ic-IV:) et 20.97% (n = 13) au stade précoce (Ia-Ib). La répartition des patientes selon les critères WHO de l'OMS était des tumeurs peu différenciées dans 22 cas (35,48%), bien différenciées dans 11 cas (17,74%), indifférenciées dans 19 cas (30,66%) et de type borderline dans 10 cas (16,13%). En situation adjuvante, 14 patientes ont bénéficié d'une chimiothérapie (22,58%) dont le protocole était dans la majorité des cas à base de sels de platine et taxane. En moyenne, le nombre de cures était égal à 3 (extrêmes: 1-9). La corticothérapie était administrée chez 6 patientes (9,68%). Les molécules utilisées ont été: methyl-prédnisolone dans 3 cas, dexaméthosone dans 2 cas et prédnisolone dans un cas. Durant la période d’étude, 5 patientes pouvaient pu bénéficier d'une radio chimiothérapie adjuvante (8.06%). Le nombre de cures variait de 1 à 4 à raison de 25 à 45 Gray pour chaque cure. Avec un recul médian de 42 mois (24-60 mois), 29 patientes (46,77%) ont été perdues de vue. L’évolution était favorable dans 17 cas (27,43%) et dans 16 cas les décès se sont survenus en postopératoire tardif (25,81%). La survie globale observée à cinq ans était de 38,71%.

**Tableau 4:** Résultats des examens histologiques

Type histologique	Effectif	Pourcentage (%)
Tumeurs séreuses et endométrioïdes	49	79,03
Tumeurs mucineuses	6	9,68
Tumeurs à cellules claires	3	4,84
Tumeurs du mésenchyme	1	1,61
Tumeurs mixtes	1	1,61
Tumeurs germinales	2	3,23
**Total**	62	100

## Discussion

Le cancer de l'ovaire est l'un des cancers pelviens les plus fréquents chez la femme [3]. Cependant, selon les registres et compte tenu des tumeurs malignes observées dans la population féminine, il s'agissait plutôt d'un cancer rare [1]. Nous n'avons observé que 5 nouveaux cas annuels depuis l'an 2000 jusqu'en 2009. En effet, ce cancer représentait 3,8% des cancers féminins en France [1] et 0,90% dans notre série. Notre résultat était toutefois assez modeste compte tenu de la monocentricité de l’étude. Au niveau mondial, il existait une grande variabilité géographique de l'incidence des cancers de l'ovaire. Le rapport est de 5 à 6 entre les pays à forte incidence et les pays de faible incidence [2, 4]. Dans les pays de l'Union Européenne, la France se situe en dessous des taux moyens d'incidence, au même rang que la Grèce, l'Italie et le Portugal. C'est dans les pays nordiques que les taux sont les plus élevés [1]. Les taux les plus bas sont retrouvés dans les pays en voie de développement [2] comme le notre (Madagascar). Le cancer de l'ovaire affecte essentiellement les femmes jeunes avec une incidence qui grimpe considérablement après 40 ans pour atteindre son point culminant au cours de la huitième décennie de vie [5]. Il est donc pour l'essentiel une tumeur de la femme ménopausée ou nullipare. En effet, dans sa forme précoce, la ménopause favoriserait la survenue d'un cancer de l'ovaire. En revanche, c'est une ménopause tardive qui serait un facteur de risque de développer un cancer au vu de la théorie de l'ovulation incessante [2]. Dans notre série, l’âge moyen des patientes était assez jeune (43 ans) comparativement aux données de la littérature (62-65 ans) [1, 6]. Cependant, nous avons constaté une prédominance des patientes âgées de plus de 45 ans (53,23%). Ceci correspond à l’âge de survenue précoce de la ménopause où le risque de développer un cancer de l'ovaire est accru [2]. Dans le même sens, il existait des patientes nullipares ou ayant eu peu d'enfants (64,52%). Il s'agissait dans la majorité des cas, des patientes à haut risque par manque de parité. Il a été rapporté que l'influence hormonale est désormais retenue avec le rôle bénéfique de la grossesse, de l'allaitement et surtout de la contraception orale [1, 2]. La prédisposition génétique classiquement retrouvée dans les tumeurs épithéliales de l'ovaire (5-10%) [2] était également observée dans notre série avec une proportion voisine (14,52%). Cependant, aucune preuve biologique oncogénétique n'a été rapportée par manque de moyens à la fois matériel et technique. Quant à l'influence hormonale, l’âge d'apparition des premières menstruations était un facteur de risque controversé et a été largement discuté [2]. A l'issu de cette revue générale, les deux principales hypothèses hormonales retenues ont été la théorie de l'ovulation incessante et l'exposition aux gonadotrophines. Le diagnostic du cancer de l'ovaire est souvent tardif, au stade avancé de carcinose péritonéale. Environ, deux tiers des patientes sont diagnostiquées à un stade IIIB ou IV [1]. Le diagnostic précoce est difficile en raison de l'absence de symptomatologie spécifique et de la mauvaise accessibilité anatomique des ovaires [7].

Les signes d'appel sont insidieux et n'apparaissent qu'en cas de tumeurs évoluées. Les symptômes évocateurs rapportés ont été: ballonnements, augmentation du volume abdominal, asthénie, troubles urinaires, douleurs pelviennes ou abdominales [2]. L'objectif des recherches était donc d’établir un diagnostic en phase infra-clinique. Ainsi, de nombreuses études ont été publiées afin de mettre en place un dépistage systématique [1, 2]. La combinaison du dosage du CA 125 avec l’échographie endovaginale a été longtemps proposée mais les résultats étaient décevants [2]. Le consensus établi n'a préconisé un dépistage que chez les femmes prédisposées [8]. Les femmes sans antécédent familial de cancer de l'ovaire n'ont pas été considérées [9]. Pourtant, les circonstances de découverte sont souvent fortuites lors des examens échographiques (87,1% dans notre série) demandés pour de multiples raisons [1, 10]. L'avantage avec l’échographie est de pouvoir suspecter la nature maligne des tumeurs ovariennes en mettant en évidence une ascite, irrégularité des contours et intrication anarchique des structures solides ou liquides de la masse tumorale. Elle permet aussi de découvrir des stades précoces mais dans le cadre de dépistage systématique. Ce qui est difficile à concrétiser en milieu défavorisé. Dans ce contexte, le CA 125 est une aide précieuse permettant de relater les données radiologiquement suspectes. En effet, il est le marqueur le plus sensible et le plus utilisé au moment du diagnostic pour évaluer la possibilité de résection complète, la sensibilité à la chimiothérapie et pour le diagnostic des récidives [11, 12]. Toutefois, sa fiabilité en prévention primaire est insuffisante puisque 50 à 60% seulement des cancers de l'ovaire stade I ont un CA 125 élevé avec une valeur prédictive positive très faible (10%) [13]. Dans notre série, seulement quelques patientes (19,35%) ont pu bénéficier de cet examen du fait de son coût élevé. En alternative, la recherche des cellules anormales dans le liquide d'ascite était proposée mais peu contributive et par conséquent moins pratiquée. A titre de bilan d'extension, la tomodensitométrie TAP, exceptionnellement faisable dans notre centre, est surtout indispensable en préopératoire si une chirurgie est envisageable ou pour surveiller l'efficacité d'une chimiothérapie de seconde ligne [12]. Par défaut, nous nous sommes contentés des examens standards tels que la radiographie du thorax et l’échographie abdominale qui peuvent mettre en évidence des lésions métastatiques respectivement au niveau pulmonaire et hépatique. Dans les centres experts sophistiqués, la détection des récidives est surtout avantageusement appréciée par le dosage du CA 125, réalisation d'un scanner TAP et TEP-TDM [12]. A part son intérêt diagnostic initial, ce dosage du CA 125 est également intéressant dans l’évaluation de la réponse au traitement.

Le seul problème avec ce marqueur est la possibilité de faux positifs observés dans de nombreuses situations gynécologiques (endométriose pelvienne, fibrome utérin, infection génitale, ovulation) et non gynécologiques (épanchement des séreuses, cirrhose, pancréatite, diverticulose) [11]. Ce manque de spécificité a motivé l’étude de «dépistage combiné» pour le cancer de l'ovaire [2]. Ce dosage est aussi source de faux négatifs dans les stades peu avancés du cancer de l'ovaire ou tumeurs borderline [11]. Actuellement, une nouvelle approche prometteuse vers une méthodologie de dépistage [13] largement applicable à la détection précoce des carcinomes gynécologiques est en cours d’évaluation [14]. En effet, des lésions préinvasives tubaires et ovariennes ont été récemment mises en évidence et devraient aider à mieux comprendre la physiopathologie des stades précoces de la cancérogenèse ovarienne [13]. Grâce au progrès de la génétique moléculaire, des gènes présents dans la plus part des cancers de l'ovaire ont été détectés et pourraient être utilisables en diagnostic. Selon une étude américaine [14], des mutations somatiques caractéristiques des cancers de l'ovaire ont été identifiées dans l'ADN purifié à partir des prélèvements de routine. Toutefois, la recherche de ces mutations ne concerne que les patientes avec un risque génétique prouvé ou dans le cadre d'une consultation d'oncogénétique [2]. Dans notre centre, cette méthode est loin d’être faisable pour des raisons financières. Le diagnostic histologique des tumeurs ovariennes ne peut être obtenu qu'en postopératoire, après exérèse des lésions observées en peropératoire. Classiquement, les tumeurs épithéliales représentaient le type histologique le plus fréquent suivies des tumeurs germinales chez les femmes jeunes [6]. La chirurgie radicale est le traitement de base à visée curative. La voie d'abord peut être menée par laparotomie (55,2%) ou par voie cœlioscopique (44,8%) en prenant le risque d'une éventuelle conversion en cas de cancers infiltrants ou aux stades avancés [3]. L'avantage avec cette chirurgie mini-invasive serait de réduire à la fois la durée opératoire et le séjour hospitalier avec une cicatrice abdominale acceptable. En intention curative, la chirurgie radicale avec chimiothérapie adjuvante est le traitement de référence dans la prise en charge des cancers de l'ovaire [12, 15]. Au vu des résultats chirurgicaux encourageants en faveur de cette chirurgie curative, les chirurgiens se sont rattachés à réaliser des chirurgies de plus en plus étendues. Actuellement, le traitement de base pour les cancers ovariens repose sur l'hystérectomie totale avec annexectomie bilatérale, omentectomie, appendicectomie, curages pelviens et lombo-aortiques avec résection de toutes les lésions macroscopiques visibles. La lympaphadénectomie pelvienne ou lomboaortique, souvent réalisée par voie transpéritonéale [3], permettait d'améliorer la survie sans récidive chez les patientes atteintes d'un cancer de l'ovaire à un stade avancé [16]. Dans notre série, toutes les patientes ont été opérées pour réduction tumorale de façon optimale. Néanmoins, l'hystérectomie n’était pas effectuée de façon systématique pour des raisons multiples: jeune âge de la patiente, nulliparité, âge assez élevé avec le risque opératoire, stade précoce ou stade trop avancé de la maladie. En effet, la chirurgie conservatrice (ovariectomie, annexectomie unilatérales) peut être proposée chez les patientes jeunes, sans enfant, et désireuses de grossesse ou porteuses d'une tumeur localisée (IA).

Toutes les séries rapportées ont démontré que si l'acte chirurgical optimal était essentiel pour parvenir à une curabilité d'une tumeur ovarienne maligne, le traitement adjuvant est indispensable afin d'améliorer le pronostic. Le traitement initial, selon les options standards et les recommandations, repose sur l'association d'un traitement chirurgical premier et d'un traitement par chimiothérapie intraveineuse à base de sels de platine et taxane [17, 18]. Des essais faits en Europe ont montré que le cancer de l'ovaire est chimiosensible [19]; l'utilisation de la radiothérapie étant diminuée au cours du temps probablement du fait du développement des molécules efficaces de la chimiothérapie [6]. Dans notre centre, la radiothérapie faisait défaut depuis le mois de janvier 2009. Le faible pourcentage de nos patientes ayant suivi ce traitement chimiothérapique adjuvant était surtout expliqué par le coût élevé des molécules recommandées, l’éloignement des domiciles et la fréquence des effets secondaires mal interprétés par les entourages avec pour conséquence abandon et refus de traitement. Pourtant, la survie des patientes opérées par chirurgie d'intervalle après chimiothérapie première était améliorée et rejoint celle des malades traitées par chirurgie initiale [20]. Dans tous les cas, les protocoles de chimiothérapie dépendent de la nature histologique de la tumeur. Cependant, l'existence de lacune en termes de faisabilité entrainait dans notre centre une modification des protocoles préconisés. Pour les tumeurs germinales et granulosa, notre protocole était basé sur le bléomycine, l'Etoposide et le Cisplatine (BEP) toutes les 4 semaines au moins 4 cycles. Pour les tumeurs épithéliales, nous avons utilisé sels de platine et taxane ou cyclophosphamide toutes les 3 semaines. Pour la corticothérapie, elle était surtout utilisée à visée palliative. A l'issue des traitements, 27,42% des patientes ont eu des suites favorables et 25,81% sont décédés. Les perdues de vue, considérées comme décédées à la date du dernier recul, étaient assez nombreuses (46,77%). Nous n'avons pas eu de contact en provenance de leur famille. Toutefois, beaucoup d'entre elles vues et traitées au stade précoce ont eu des résultats cliniques favorables après chirurgie radicale première et quelques cures de chimiothérapie avec un recul supérieur à 4 ans (11,29%). Dans cette série, la survie globale observée à cinq ans était proche de celle rapportée par Benoit et al entre 1982 et 1996 [6]. Dans les formes avancées, le taux de mortalité reste élevé en absence de chirurgie radicale et de chimiothérapie intra-péritonéale (CIP) [21]. En cas de récidive, la chimiothérapie hyperthermique intra-péritonéale (CHIP) a été proposée en toute sécurité afin d'améliorer la survie des patientes avec une morbidité acceptable et des résultats encourageants [12]. Dans notre série, elle n'a pas été proposée par manque d'expérience. La mortalité (25,81%) était surtout alourdie par le retard diagnostique, le coût inaccessible du traitement adjuvant et la non observance thérapeutique.

## Conclusion

Le cancer de l'ovaire n’était pas fréquent mais grave compte tenu des stades avancés observés au moment du diagnostic et du taux élevé des patientes décédées par manque de traitement adjuvant adapté. Cette étude nous a permis de dégager les particularités épidémio-cliniques, diagnostiques et thérapeutiques du cancer de l'ovaire pris en charge dans un pays à faibles ressources. Le retard diagnostic, le manque de dépistage, le coût élevé du CA 125, et les difficultés d'accès aux moyens thérapeutiques sophistiqués étaient à l'origine de nos résultats décevants malgré le progrès des efforts techniques apporté par la chirurgie conventionnelle à visée curative. Ces difficultés de prise en charge pourraient être remédiées par la mise en œuvre d'une méthodologie de dépistage accessible en milieu défavorisé. Le progrès de la biologie moléculaire applicable à la détection précoce des carcinomes gynécologiques pourrait être un atout pour les centres mieux équipés.
